# Draft Genome Sequence of *Pantoea ananatis* GB1, a Plant-Growth-Promoting Hydrocarbonoclastic Root Endophyte, Isolated at a Diesel Fuel Phytoremediation Site Planted with *Populus*

**DOI:** 10.1128/genomeA.00028-16

**Published:** 2016-02-25

**Authors:** Panagiotis Gkorezis, Jonathan D. Van Hamme, Eric M. Bottos, Sofie Thijs, Maria Balseiro-Romero, Carmela Monterroso, Petra Suzan Kidd, Francois Rineau, Nele Weyens, Jaco Vangronsveld

**Affiliations:** a Hasselt University, Centre for Environmental Sciences, Diepenbeek, Belgium; b Thompson Rivers University, Department of Biological Sciences, Kamloops, Canada; c University of Santiago de Compostela, Department of Soil Science and Agricultural Chemistry, Santiago de Compostela, Spain; d Instituto de Investigaciones Agrobiológicas de Galicia (IIAG), Consejo Superior de Investigaciones Científicas (CSIC), Santiago de Compostela, Spain

## Abstract

We report the 4.76-Mb draft genome of *Pantoea ananatis* GB1, a Gram-negative bacterium of the family *Enterobacteriaceae*, isolated from the roots of poplars planted for phytoremediation of a diesel-contaminated plume at the Ford Motor Company site in Genk, Belgium. Strain GB1 promotes plant growth in various hosts and metabolizes hydrocarbons.

## GENOME ANNOUNCEMENT

The genus *Pantoea* is known for its metabolic versatility (1), and *P. ananatis* has been globally implicated as a pathogen in significant agricultural crops and forest tree species (2). More recently, *P. ananatis* strains have been reported to exhibit a range of host interactions including beneficial, weakly pathogenic, commensal, and mutualistic (3, 4). We found that *P. ananatis* GB1 promotes the growth of hybrid poplar cuttings (*Populus deltoides* × [*trichocarpa* × *deltoides*] cv. Grimminge), as well as *Lupinus luteus* and *Cytisus striatus*, even though 16S rRNA gene sequence data along with phenotypic profiling indicate that GB1 is closely related to the rice pathogen *Pantoea ananatis* PA13 (5).

An Ion Torrent PGM (Life Technologies, Inc., Carlsbad, CA) was used for sequencing after extracting DNA from stationary-phase cells of GB1 using standard techniques as described by Thijs et al. (6). In total, 1.52 million reads (mean length 265 bases) generated 403 Mb of data (>362 M Q20 bases) in Torrent Suite 4.2.1. These were assembled using SPAdes 3.1.0 (7, 8) (uniform coverage mode; k-mers 21, 33, 55, 77, 99) into 33 contigs greater than 500 bp, giving a consensus length of 4,765,050 bp at 51.0× coverage (largest contig 780,856 bp; *N*_50_ = 363,336 bp). The Rapid Annotations using Subsystems Technology (RAST) server (9) was used to identify closely related organisms with complete genomes in NCBI RefSeq. *Pantoea ananatis* PA13 (GenBank accession no. CP003085) was used as a reference to reorder the GB1 contigs in Mauve (10).

Genome annotation was completed in the PGAP (NCBI) pipeline (11). The *P. ananatis* GB1 genome is 4.76 Mb and consists of one chromosome (55.2 % G+C) which includes 4,433 coding genes that were arranged into pathways using Pathway Tools (12, 13), 273 pseudogenes, 13 rRNAs (5S, 16S, 23S), 53 tRNAs, and 6 noncoding RNAs (ncRNAs).

Homologues of genes related to plant growth promoting traits such as inorganic phosphorus solubilization, nitrogen fixation, and siderophore production were found, along with multiple copies of genes for alkanes utilization such as: alkane monooxygenase genes (*alkB*), medium-chain acyl-CoA dehydrogenase (*acdA*) genes, and long-chain acyl-CoA dehydrogenase (*acdB*) genes. Interestingly, GB1 carries a bacterial laccase gene, in agreement with reports of a novel bacterial laccase, Lac4, produced by *P. ananatis* Sd-1, a strain that is able to oxidize nonphenolic and phenolic compounds (14).

The use of *P. ananatis* GB1 plant-growth-promoting inoculant for improving phytoremediation of diesel-contaminated sites is being explored.

### Nucleotide sequence accession numbers.

This whole-genome shotgun project has been deposited at DDBJ/EMBL/GenBank under the accession number JYGW00000000. The version described in this paper is version JYGW01000000.
